# Prognostic Significance of CK5/6 and GATA3 Expression in Recurrent Urothelial Carcinoma

**DOI:** 10.3390/cancers17193267

**Published:** 2025-10-09

**Authors:** Marzena Lenda-Petrykowska, Violetta Sulżyc-Bielicka, Wojciech Dobrzycki, Krzysztof Safranow, Jerzy Świtała, Dorota Kostrzewa-Nowak, Paweł Bielicki

**Affiliations:** 1 Department of Vascular, General and Angiological Surgery, University Clinical Hospital No.2, Pomeranian Medical University in Szczecin, 72 Powstańców Wlkp. Al., 70-111 Szczecin, Poland; maszajn@gmail.com; 2 Department of Medical Oncology, Pomeranian Medical University in Szczecin, 4 Arkońska St., 71-455 Szczecin, Poland; 3 Department of Pathomorphology, Provincial Specialist Hospital in Szczecin, 4 Arkońska St., 71-455 Szczecin, Poland; 4 Department of Biochemistry and Medical Chemistry, Pomeranian Medical University in Szczecin, 72 Powstańców Wlkp. Al., 70-111 Szczecin, Poland; 5 Department of Urology, Provincial Specialist Hospital in Szczecin, 4 Arkońska St., 71-455 Szczecin, Poland; 6 Department of Clinical and Molecular Biochemistry, Pomeranian Medical University in Szczecin, 72 Powstańców Wlkp. Al., 70-111 Szczecin, Poland; 7 Department of Radiotherapy, Pomeranian Medical University in Szczecin, 22 Strzałowska St., 71-730 Szczecin, Poland; pawelbielicki68@gmail.com

**Keywords:** urothelial carcinoma, CK5/6, GATA3

## Abstract

Background/Objectives: The study aimed to determine whether the expression of CK5/6 and GATA3 is altered in UCa recurrences and to evaluate disease-free survival (DFS) and overall survival (OS) according to CK5/6 and GATA3 expression. Methods: A retrospective study was performed in 77 patients with UCa. Surgery was performed in 35 patients. UCa recurrence was observed in 75% of patients. An immunohistochemical assessment of CK5/6 and GATA3 was performed in the primary and recurrent UCa groups. Results: CK5/6(+) in primary UCa was associated with a 73% probability of CK5/6(+) recurrence (*p* = 0.000005) and incidence at a younger age. CK5/6(−) in primary UCa was associated with an 84% probability of CK5/6(−) recurrence (*p* = 0.000005) and incidence at older age. A higher probability of UCa GATA3(+) recurrence was a significant independent factor associated with longer OS (*p* = 0.015). A greater probability of UCa CK5/6(+) recurrence was a significant independent factor associated with shorter OS (*p* = 0.044). Patients with CK5/6(+)-only UCa recurrences had significantly worse OS compared to UCa patients with at least one CK5/6(−) recurrence. Conclusions: 1. CK5/6 and GATA3 in UCa recurrences may differ from CK5/6 and GATA3 expression in primary UCa. Intensified oncological surveillance is suggested for patients with recurrent CK5/6(+) 2. Patients with at least one UCa CK5/6(−) recurrence have better prognosis compared to patients with only CK5/6(+) recurrences.

## Simple Summary

This study aimed to assess the expression of CK5/6 and GATA3 alterations in urothelial carcinoma (UCa) recurrences and evaluate overall survival (OS) and disease-free survival. A retrospective study was performed in 77 patients. CK5/6 and GATA3 were assessed immunohistochemically in primary and recurrent UCas. Primary CK5/6(+) UCa was associated with a 73% probability of recurrent CK5/6(+) (*p* = 0.000005) and incidence at younger age. Primary CK5/6(−) UCa was associated with an 84% probability of CK5/6(−) recurrence (*p* = 0.000005) and incidence at older ages. A higher probability of UCa CK5/6(+) recurrence was a significant independent factor associated with shorter OS (*p* = 0.044). A higher probability of UCa GATA3(+) recurrence was a significant independent factor associated with longer OS (*p* = 0.015). The evaluation of CK5/6 and GATA3 in recurrent UCa allows the early assessment of prognosis and appropriate oncological surveillance.

## 1. Introduction

Urothelial carcinoma is one of the most common malignant neoplasms in humans. The countries with the highest incidence of UCa include the United States and European countries [1,2,3,4,5,6,7,8]. The most significant risk factor for UCa is smoking [1]. The following factors have been proven to also influence the development of urothelial carcinoma: exposure to aromatic amines, use of cyclophosphamide, drinking water contaminated with arsenic, chlorides and pesticides, and eating nitrite-processed meat [1,2,3,4,5,6]. The risk of developing UCa in smokers increases with the number of pack-years and is 3–4 times higher than in non-smokers [1,2,3,4,5,6,7,8]. It is worth noting that the history of radiation therapy to the pelvic area is associated with an increased risk of developing UCa [2,3,7,8]. Moreover, the occurrence of endemic Schistosoma haematobium infections in some regions of Asia and Africa is associated with an increased risk of developing bladder cancer (BC) [1,2,3,6,7,8].

UCa is most commonly diagnosed in the bladder, in about 90% of cases [2,3,5,7,8], but it can develop in both the upper and lower urinary tract [2,3,7,8,9] because both are covered by the same type of epithelium, called transitional epithelium.

Depending on the involvement of the bladder’s muscularis propria, UCas are classified as non-muscle-invasive bladder cancer (NMIBC) or muscle-invasive bladder cancer (MIBC) [7,8]. NMIBCs include Ta, Tis, and T1 tumors; MIBCs include T2, T3, and T4 tumors [2,7].

Nearly 80% of BCs are superficial papillary lesions developing from papillary hyperplasia and low-grade neoplasia. These lesions can be multifocal and often recur after excision, but relatively rarely become invasive [4]. Other BCs are infiltrative lesions that are non-papillary, develop from high-grade neoplasia or carcinoma in situ (CIS), and have aggressive clinical course [4]. Depending on histological grade, UCas can be classified as low-grade (G1, G2) and high-grade (G3) [2,3,8]. The five-year survival rate at the Ta, Tis, and T1 stages is estimated at 70%, while at the T3 and T4 stages, it has been estimated at less than 20% [2,3,8].

UCas can spread through the physiological pathway of urine outflow into the bladder. Cystoscopy and TURB procedures can lead to micrometastases [2,3,8]. Sixty to eighty percent of patients are found to have disease confined to the bladder at diagnosis. Distant metastases are present in 10–20% of cases [2,3,4,8]. Urothelial carcinoma tends to be recurrent, and the classification of infiltrating and non-infiltrating tumors impacts therapeutic decisions.

NMIBC treatment involves radical local resection with transurethral resection of bladder tumor (TURBT) [2,3,8]. Patients with NMIBC are classified as having a low, intermediate, or high probability of recurrence and disease progression according to the AUA (American Urological Association)/EUA (European Association of Urology) scale [2,3,8]. The high-risk group, most prone to tumor progression, includes T1G3 cancers, with tumor diameter >3 cm, high grade, CIS (carcinoma in situ), recurrent tumors, localized within the prostatic urethra, with rare histologic types, lymphatic and blood vessel involvement [2,3,8].

The treatment of choice for patients with BC of at least the T2 stage is radical cystectomy with pelvic lymphadenectomy [2,3,8].

Czerniak et al. [4] distinguished two paths of bladder cancer development: 1. based on papillary lesions, which occur in more than 70% of BC and has the character of recurrent non-infiltrating lesions. 2. based on non-papillary lesions of high-grade intraepithelial neoplasia, which tend to infiltrate and metastasize [4].

In recent years, based on the results of mRNA profiling, new classifications of infiltrating UCa have been proposed, which impact urothelial cancer prognosis [4,10,11,12,13].

The results of mRNA profiling have been compared with those of immunohistochemical (IHC) evaluation on the expression of proteins significant for UCa development. IHC studies highly correlate with genetic assays [4,10,11,12,14,15,16]. It should be noted that immunohistochemical testing is widely available, inexpensive, and can be performed on archival material in most pathomorphology departments. Assessment of GATA3 and CK5/6 is considered sufficient for classifying MIBC into primary subtypes [17,18,19].

GATA3 is a transcription factor involved in epithelial cell differentiation. It is involved in embryogenesis, humoral and inflammatory responses. Down-regulation of GATA3 expression promotes neoplastic transformation [19]. GATA3 immunostaining is used as a diagnostic marker for breast and urothelial cancer but it can also occur in other neoplasms. CK5/6 is an alpha-type polypeptide filament found in the cytoskeleton of epithelial cells [19].

Distinct IHC “markers” characterize the different subtypes: luminal GATA3(+), CK5/6(−) and basal GATA3(−), CK5/6(+) [4,12,15,20]. Of the remaining group of non-basal and non-luminal tumors, “double-negative” (negative staining for GATA3 and CK5/6) and mixed (positive staining for GATA3 and CK5/6) tumors are distinguished [4,20]. UCa subtypes differ in histological structure, clinical course, and sensitivity to chemotherapy [4,15,17,20,21,22,23,24,25,26].

Luminal carcinomas present high expression of GATA3, CK20, and uroplakin 2, which are markers of terminal differentiation of urothelial cells [4,16,19]. Basal carcinomas present expression of mesenchymal cell biomarkers CK5/6 and CK14 and features of sarcomatoid tumors [4,23]. Basal cell carcinomas are diagnosed at more advanced stages and have an aggressive clinical course, lower histologic maturity, and worse overall survival [4,15,27]. Some reports suggest that the basal subtype is associated with a better response to chemotherapy than the luminal UCa subtype [4]. Basal cell carcinomas, according to some reports, are more common in women [4,28].

This study aimed to determine whether expression of CK5/6 and GATA3 is altered in UCa recurrences and evaluate disease-free survival (DFS) and overall survival (OS) according to CK5/6 and GATA3 expression.

## 2. Material and Methods

This study was approved by the Bioethics Committee of Pomeranian Medical University, No. KB-0012/237/06/18.

A retrospective study was conducted on an unselected cohort of 77 patients diagnosed with UCa of the bladder or ureter, without distant metastases. All patients were treated at the Departments of Clinical Oncology and Urology at the Provincial Specialist Hospital in Szczecin between 2013 and 2021. At the time of diagnosis, the mean age of the patients was 68.9 years (min.: 49; max: 86; median: 68). The study cohort included 17 women (22.1%) and 60 men (77.9%; Table 1).

The diagnosis of UCa was established based on cystoscopic imaging results, confirmed via histopathological evaluation of the biopsies. Tissue samples, collected during subsequent cystoscopies, were evaluated in the Department of Pathomorphology of Provincial Specialist Hospital in Szczecin. The staging of the tumor and grading of the UCa were evaluated.

UCa biopsies taken during cystoscopy that did not involve mucosal muscularis propria were evaluated as Tx (Table 1). In the case of surgical treatment, it was possible to evaluate pT and pN features in the histological material. Patients with ≥ T2 or ≥ T1G3 tumors qualified for surgery.

Surgical treatment was performed in 35 patients (45.4%). Cystectomy was performed in thirty-one patients (40.3%), while nephrectomy was performed in four patients (5.2%). Forty-two patients (54.5%) were not treated surgically. Eighteen patients (23.4%) were disqualified from surgery due to medical contraindications. Surgical treatment was performed in the Department of Urology. In female patients, cystectomy involved complete removal of the bladder, urethra, uterus, adnexa, and the anterior vaginal wall. In male patients, cystectomy included excision of the bladder, prostate, and seminal vesicles. Nephroureterectomy consisted of resection of the kidney, renal pelvis, and entire ureter. Urological follow-up included physical examination, abdominal ultrasonography, and cystoscopic evaluation.

Chemotherapy was administered at the Clinical Oncology Department. Forty-six patients were qualified for chemotherapy, including 30 patients for neoadjuvant chemotherapy and 12 patients for adjuvant chemotherapy. Four patients received palliative chemotherapy. Patients with ≥ T2 tumors were qualified for neoadjuvant chemotherapy. Patients who had not previously received neoadjuvant chemotherapy were qualified for adjuvant chemotherapy in pN(+) or ≥pT2 tumors. The regimens used for neoadjuvant or adjuvant treatment included cisplatin and gemcitabine. Carboplatin in combination with gemcitabine was used for palliative chemotherapy.

Gemcitabine was administered at a dose of 1000 mg per m2 of body surface area via 30 min intravenous infusions on days 1, 8, and 15 of each 28-day treatment cycle in combination with cisplatin or carboplatin. Cisplatin was administered at doses of 70 mg per m2 of body surface area on day 2 of the cycle. If carboplatin was chosen (in place of cisplatin), drug doses were calculated based on Calvert’s formula, where dose [mg] = AUC × (eGFR + 25), assuming AUC = 4.5 or 5, and administered via intravenous infusions lasting approximately 60 min. Chemotherapy cycles were administered every 3–4 weeks. Before administering a cycle of chemotherapy, patients underwent a physical examination and laboratory tests were performed.

Radiation therapy was used in 18 (23.4%) patients who were disqualified from surgery or as a palliative treatment. Irradiation combined with chemotherapy or radiotherapy alone used a total dose of 64–66 Gy in 32–33 fractions. In palliative treatment, the doses used were 20 Gy in 5 fractions or 30 Gy in 10 fractions.

**Figure 1:**
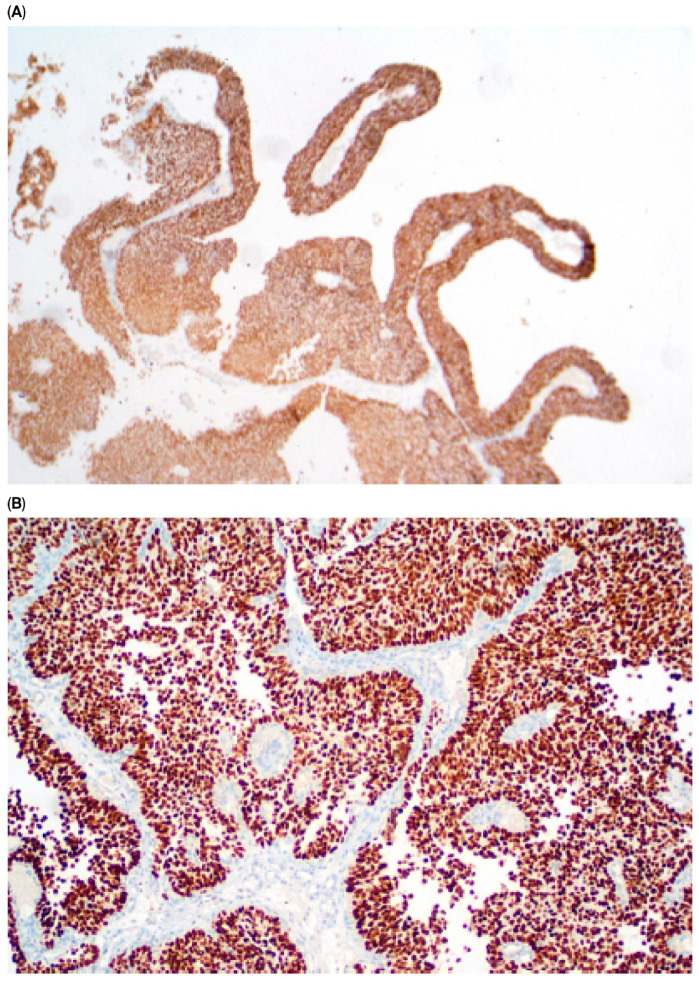
Positive GATA-3 reaction in primary UCa, magn. ×40 (**A**) and in recurrent UCa, magn. ×100 (**B**).

**Figure 2:**
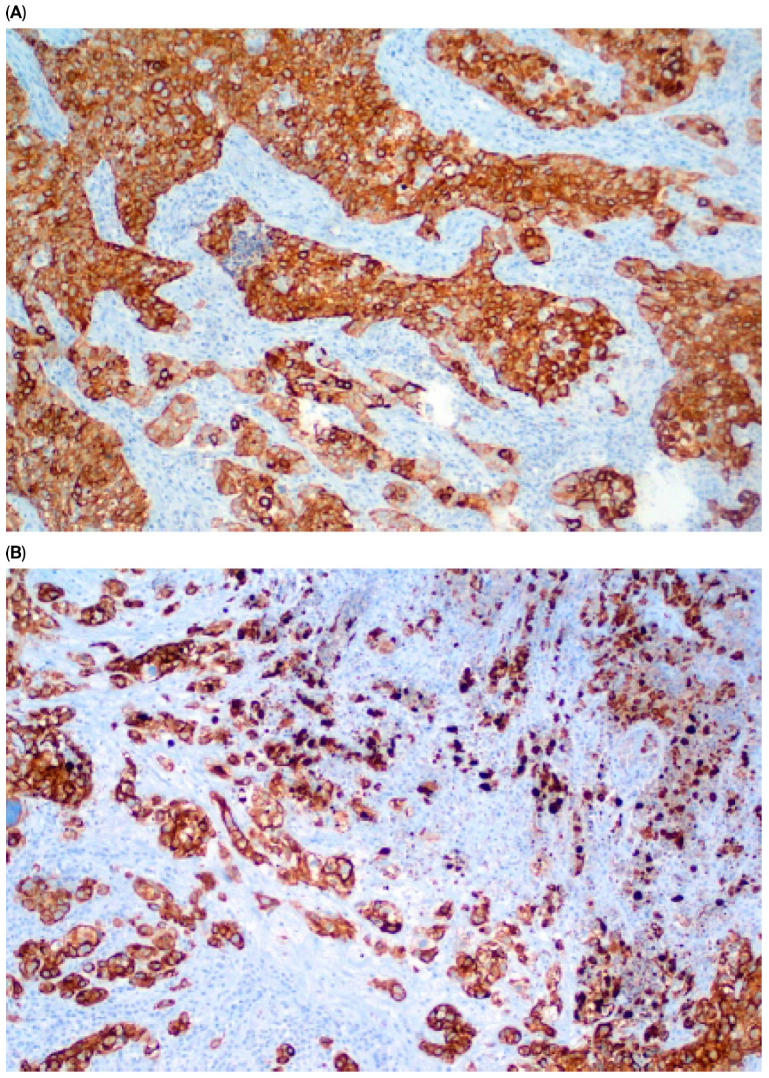
Positive CK5/6 reaction in primary UCa, magn. ×100 (**A**) and in recurrent UCa, magn. ×100 (**B**).

**Figure 3:**
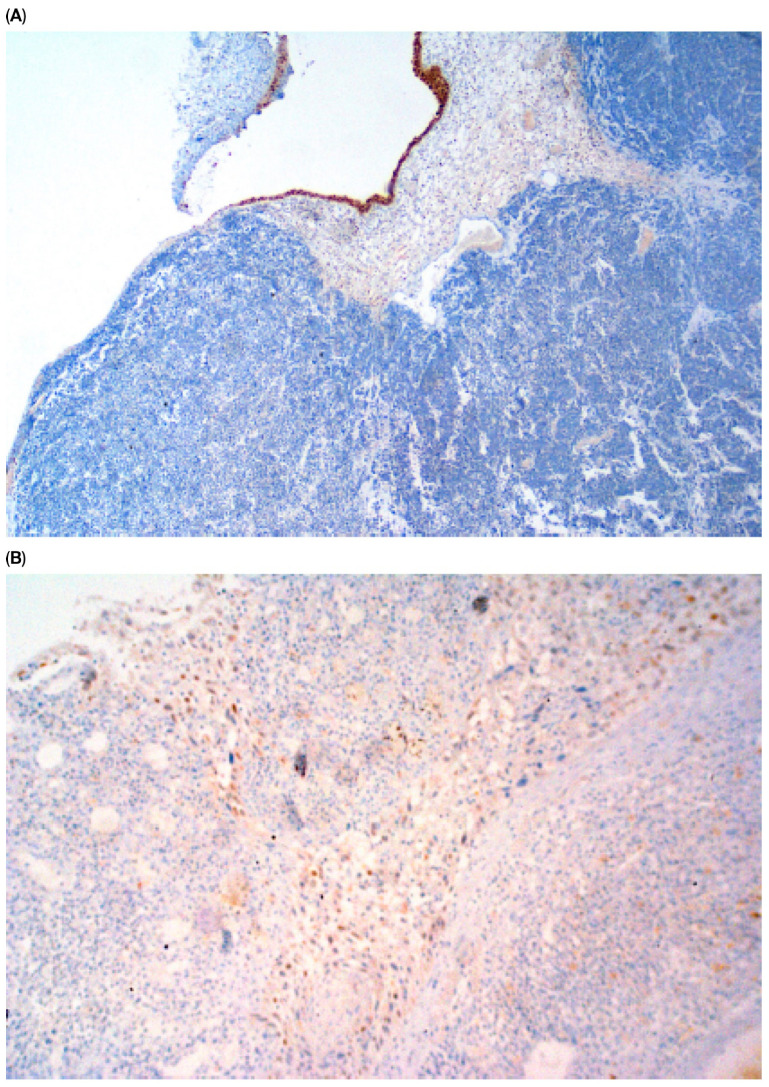
Negative GATA-3 reaction in primary UCa, magn.×40 (**A**) and in recurrent UCa, magn ×100 (**B**).

**Figure 4:**
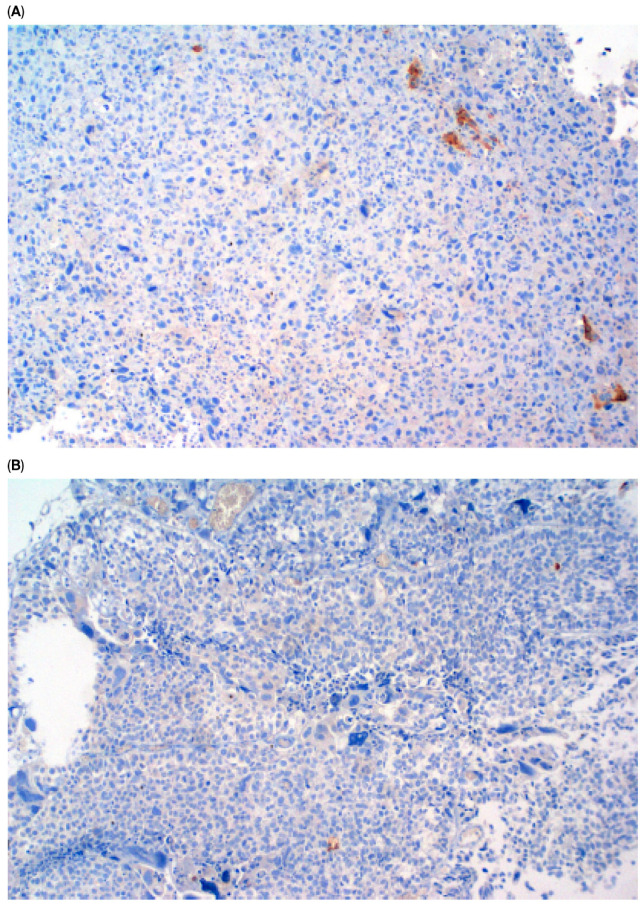
Negative CK5/6 reaction in primary UCa, magn. ×100 (**A**) and in recurrent UCa, ×100 (**B**).

Immunohistochemical staining (IHC) of CK5/6 and GATA3 expression was performed on UCa histopathological material. Tumor tissue was fixed in buffered 10% formalin and embedded in paraffin. Roche monoclonal GATA-3 antibody (L50-823; dilution ready to use: 2.34 µg/mL; incubation time: 20 min) and monoclonal Cytokeratin 5/6 antibody (D5/16B4; dilution ready to use: 5.6 µg/mL; incubation time: 20 min) were used. Immunohistochemical staining was performed in an automated Roche Ventana BenchMark GX. A peroxidase–antiperoxidase detection system was used. The reaction was developed with a diaminobenzidine substrate—chromogen solution and slides were counterstained with hematoxylin. Appropriate internal positive and negative controls were assessed. Strong, dark brown nuclear (GATA3) and cytoplasmic (CK 5/6) stains were accepted as positive. All stains were assessed by one pathologist with over 30 years ’experience in immunohistochemistry. Representative images of positive and negative immunohistochemical reactions for GATA3 and CK5/6 are shown in Figure 1, Figure 2, Figure 3 and Figure 4, respectively. Due to poor quality of histological material, immunohistochemical staining could not be performed in 15 of 224 (6.7%) tumors (total primary and recurrent UCa).

The examined specimens were collected prior to systemic treatment, in both neoadjuvant and adjuvant treatment.

In our study, UCa was divided into four subtypes depending on the expression of GATA3 and CK5/6: Subtype1: GATA 3(+), CK 5/6(−), luminal subtype; Subtype2: GATA3(−), CK 5/6(+), basal subtype; Subtype3: GATA3(+), CK 5/6(+), mixed subtype; Subtype4: GATA3(−), CK5/6(−), double-negative subtype.

Luminal carcinomas were tumors, where GATA-3 staining was present in at least the superficial layers of the urothelium. They usually encompassed most of the volume of the neoplastic urothelium. Basal carcinomas presented Cytokeratin 5/6 staining in urothelial cells above the basal layer; reactions in the adjacent non-tumor-lesioned urothelium were considered as a control.

Due to the small group of patients with basal subtype UCa (*n* = 2) and double-negative cancer (*n* = 2), no survival analysis was performed for UCa subtypes. The survival analysis included the single immunohistochemical markers GATA3 and CK5/6.

Disease-free survival (DFS-1) was evaluated using data from medical records. For the purposes of this study, DFS-1 is the time from first diagnosis to recurrence ≥T1. Overall survival was analyzed. OS was defined as the time from first cancer diagnosis to death or last follow-up.

The probability of recurrence with GATA3(+), GATA3(−), CK5/6(+), and CK5/6(−) expression was assessed in each patient; the ratio of the number of recurrences with a given expression (as above) in the specimens to the number of all recurrences in which CK5/6 and GATA3 expression was assessed. The probability of recurrence with CK5/6 expression and that with GATA3 expression were assessed via statistical analyses of overall survival and disease-free survival.

The distributions of most of the quantitative variables differed significantly from the normal distribution (Shapiro–Wilk test), so their values were compared between groups using the non-parametric Mann–Whitney U test. Survival curves were analyzed using the Kaplan–Meier method, and survival time was compared between groups using the log-rank test. The association of endpoint achievement hazards (relative hazard (HR) along with 95% confidence interval (95% CI)) with clinical and molecular parameters was analyzed using the univariate and multivariate Cox proportional hazards model. The threshold for statistical significance was *p* < 0.05, and statistical calculations were performed using Statistica 13.

## 3. Results

Among the patients examined, there were 4 cases of upper urinary tract cancer (UTUC) and 73 cases of bladder cancer.

The tumor stages (T) and grades (G) of UCa at the time of diagnosis are shown in Table 1.

**Figure 5:**
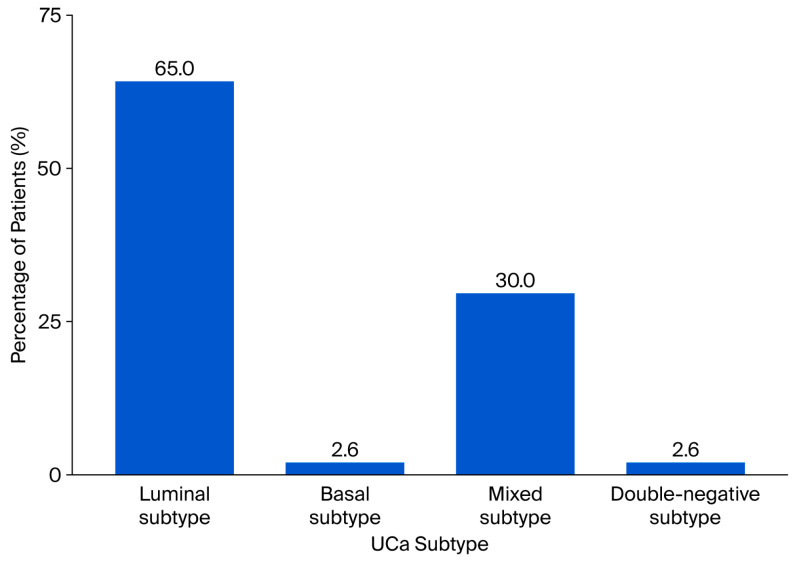
Percentage distribution of UCa subtypes in primary tumors.

For 77 patients, tumor subtype was evaluated in primary UCa samples. Subtype 1—luminal was found in fifty patients (64.9%); subtype 2—basal, in two patients (2.6%); subtype 3—mixed, in twenty-three (30.0%) patients; and subtype 4—double-negative, in two (2.6%) patients (Figure 5).

A positive IHC CK5/6 reaction was found in 25 (32.5%) primary tumors.

A positive IHC GATA3 reaction was found in 73 (94.8%) primary tumors.

Of the 77 (100%) patients, 58 (75.32%) patients had subsequent recurrences.

The frequency of positive and negative IHC reactions for GATA3 and CK5/6 is presented in Table 2.

Tumor location in upper urinary tract (UTUC) vs. BCa was not associated with expressions GATA3 and CK5/6 in the primary tumor and in recurrences (respectively, for GATA3, *p* = 1.000, and for CK5/6, *p* = 0.592).

No immunohistochemical staining available for 1 patient diagnosed with first recurrence.

No recurrence of urothelial carcinoma (UCa) was observed in *n* = 19 (24.7%) patients.

Grading was evaluated in the available histopathological material. As shown in Table 3, 27 cases did not have their grade evaluated due to insufficient quality of histopathological samples.

Using the Mann–Whitney U test, UCa CK5/6(−) patients were compared to CK5/6(+) patients in the primary tumor. Mean age at diagnosis, probability of UCa recurrence without CK5/6(−) expression, and the probability of UCa recurrence with CK5/6(+) expression were compared in both groups (Table 4).

There was a significant association of CK5/6 expression in primary UCa with expression in recurrences and with the age of patients at diagnosis. Urothelial carcinomas with CK5/6(+) expression in the primary tumors samples have been associated with a significantly younger age of diagnosis (mean age: 66 years). Primary UCa without CK5/6 (-) expression has been associated with older age at diagnosis (mean age: 70 years, *p* = 0.046, Table 4). Primary UCa without CK5/6(−) expression was associated with an 84% probability of recurrence without CK5/6(−) expression and a 15% probability of recurrence with CK5/6(+) expression (*p* = 0.000005). Primary UCa with CK5/6(+) expression was associated with a 27% probability of recurrence without CK5/6(−) expression and a 73% probability of recurrence with CK5/6(+) expression (*p* = 0.000005, Table 4).

It was not possible to demonstrate an association of GATA3 expression in the primary tumors with GATA3 expression in UCa recurrences, due to the small number of patients with urothelial carcinoma without GATA3(−) expression.

### 3.1. Survival Analysis

#### Disease-Free Survival (DFS)

The mean follow-up time of DFS-1 was 9.31 months (min.0–max.64).

### 3.2. Univariate Analysis

Radical surgery was associated with significantly shorter DFS-1. In univariate analysis, shorter DFS-1 was influenced at the limit of statistical significance by a higher T stage at diagnosis (*p* = 0.061). Longer DFS-1 was associated at the limit of statistical significance with the presence of GATA3(+) expression (*p* = 0.071) (Table 5).

Neoadjuvant chemotherapy was not associated with GATA3 and CK5/6 expression in the primary tumor and recurrence (respectively, *p* = 0.289 and *p* = 0.293), nor with OS (*p* = 0.982), but was associated with longer (lower risk) DFS-1 (*p* = 0.041, Table 5). This means that there is no data for the interaction of neoadjuvant chemotherapy with expression of GATA3 and CK5/6 in terms of its impact on survival, because DFS was associated with neoadjuvant chemotherapy but not with expression GATA3 and CK5/6.

Tumor location (UTUC vs. BCa) was not associated with survival (DFS-1, *p* = 0.756).

### 3.3. Overall Survival (OS)

The mean OS was 38.30 months (min.5–max.96), and death occurred in 37 patients (48.0%).

### 3.4. Univariate Analysis

**Figure 6:**
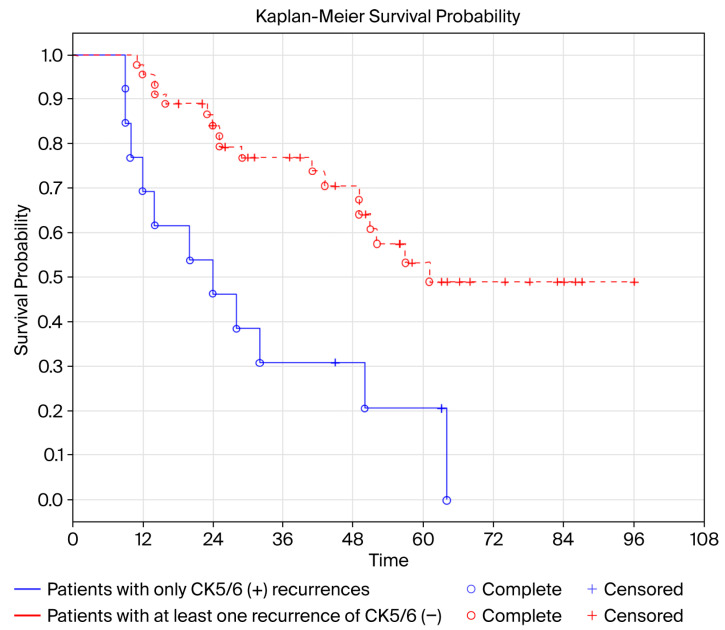
Probability of OS in patients stratified according to the number of recurrences with CK5/6(−) expression (at least one recurrence with CK5/6(−) expression vs. only CK5/6(+) recurrences). Log-rank test. *p* = 0.00327. Kaplan–Meier curves.

A higher probability of recurrence with CK5/6(+) expression was associated with significantly shorter OS (*p* = 0.004), while a higher probability of recurrence with GATA3(+) expression was associated with significantly longer OS (*p* = 0.026, Table 6, Figure 6). Tumor location (UTUC vs. BCa) was not associated with OS (*p* = 0.370).

The Kaplan–Meier OS curve of patients with only CK5/6(+) recurrences was significantly worse compared to patients with at least one CK5/6(−) recurrence (Log-rank test, *p* = 0.00327, Figure 6).

### 3.5. Multivariate Analysis

Multivariate analysis (*n* = 43) included age, sex, surgery, tumor grade in primary UCa, primary tumor stage T, and probability of recurrence GATA3(+) and CK5/6(+) (Table 7).

A higher probability of CK5/6(+) recurrence was a significant independent factor associated with shorter OS (*p* = 0.044). In contrast, a higher probability of recurrence with GATA3(+) expression was a significant independent factor associated with longer OS (*p* = 0.015, Table 7). OS was associated with expression GATA3 and CK5/6, but not with neoadjuvant chemotherapy. Therefore, we decided not to include neoadjuvant chemotherapy in the OS multivariate model (Table 7).

### 3.6. Summary of Results

Urothelial carcinomas with CK5/6(+) expression in the primary tumors were associated with a 73% probability of CK5/6(+) recurrence (*p* = 0.000005) and incidence at a younger age.

Urothelial carcinomas without CK5/6(−) expression in the primary tumors were associated with an 84% probability of recurrence without CK5/6(−) expression (*p* = 0.000005) and incidence at older age.

A higher probability of recurrence with GATA3(+) expression of urothelial carcinomas was associated with longer OS (*p* = 0.015).

A higher probability of UCa recurrence with CK5/6(+) expression was associated with shorter OS (*p* = 0.044). Patients with CK5/6(+)-only UCa recurrences had significantly worse OS compared to UCa patients with at least one CK5/6(−) recurrence.

Neoadjuvant chemotherapy was associated with longer DFS-1 (*p* = 0.041).

## 4. Discussion

In recent years, new subtypes of UCa have been identified based on genetic profiling and immunohistochemical evaluation of selected tumor proteins [4,10,11,12,14,15,16]. It has been demonstrated that immunohistochemical methods can be used in place of expensive and less accessible molecular tests [15,17,19,20,23,29,30]. Recent studies suggest that GATA3 and CK5/6 expression can identify molecular subtypes in 80–90% of cases [23]. Most researchers distinguish two fundamental subtypes of UCa: luminal with positive GATA3 expression and basal with positive CK5/6 expression [4,23].

The clinical course of urothelial carcinoma is characterized by its frequent recurrences. The present study aimed to determine whether the expression of CK5/6 and GATA3 changes in urothelial carcinoma recurrence compared to primary UCa. In our study, primary UCa without CK5/6(−) expression was associated with an 84% probability of recurrence without CK5/6(−) expression and a 15% probability of recurrence with CK5/6(+) expression (*p* = 0.000005). Urothelial cancers with CK5/6(+) expression in primary tumors were associated with a 27% probability of recurrence without CK5/6(−) expression and a 73% probability of recurrence of cancer with CK5/6(+) expression (*p* = 0.000005).

In the present study, the association of GATA3 expression in primary tumors with GATA3 expression in urothelial carcinoma recurrences could not be assessed due to the small number of UCa patients without GATA3(−) expression in primary UCa.

There are no reports in the available literature evaluating the association of specific UCa subtypes and GATA3 and CK5/6 expression in the primary tumor in association with subsequent recurrence. To our knowledge, this is the first report that compares the expression of CK5/6 and GATA3 in primary UCa and in UCa recurrences and evaluates disease free survival and overall survival according to CK5/6 and GATA3 expression. Pena et al., with a group of 38 patients, after cystectomy and pelvic lymph node dissection, described the association of molecular changes in the primary tumor with unilateral lymph node (LN) metastasis [31]. The authors define luminal tumors as the most stable. Ninety percent of metastases of the luminal subtype of UCa in LN also belonged to the luminal subtype. The basal subtype in primary UCa tumor was associated with basal subtype in metastases in the LN in 60% of cases, and in the remaining 40%, metastases in the LN were classified as luminal or mixed UCa subtypes. Patients with mixed subtype in the UCa primary tumor had both luminal and basal cancer subtypes in metastatic LNs [31]. Ravanini et al. [17] also found only moderate agreement between the immunohistochemical subtypes identified in bladder tumors and their lymph node metastasis in a study of surgical material from 183 patients with UCa who underwent cystectomy. Sjödahl et al. [32] assessed the concordance of molecular subtypes in 67 pairs of MIBC and synchronous metastases in the LN, using IHC and mRNA profiling for 57 BC and 28 matched metastases in the LN. These authors showed that discordant subtype classification between BC and in metastases in the LN was not frequent (18%), but most (58%) involved the basal/squamous-like subtype [32]. Recently, in a group of 199 patients with MIBC undergoing cystectomy, Olah et al. also demonstrated subtype heterogeneity between primary tumors and LN metastases [33]. Comparing the primary MIBC and LN metastatic sites, the authors observed an overall consistency of 57% [33]. The basal subtype exhibited high stability both within the tumors and between the primary tumors and matched positive LNs [33].

Using the Mann–Whitney U test, we compared UCa CK5/6(−) patients to CK5/6(+) patients in primary tumors. The analysis included the mean age at diagnosis and probability of CK5/6(−) and CK5/6(+) recurrence. We demonstrated that urothelial carcinomas with CK5/6(+) expression in the primary tumors were associated with significantly younger age at diagnosis (mean age, 66 years). Patients with UCa without CK5/6(−) expression in the primary UCas were older at the time of diagnosis (mean age, 70 years).

Some studies indicate that CK5/6(+) expression in MIBC is an “unfavorable” prognostic marker and is associated with shorter survival times [14,27,34]. Hashimi et al., in a group of 127 tumors, did not show an association of CK5/6 expression with OS and DFS [26].

In a study based on immunohistochemical evaluation of GATA3, CK5/6, and CK5, Serag Eldien et al. suggested an association between molecular subtype and better prognosis in luminal MIBC [18]. According to most reports, patients with mixed and basal UCa have an aggressive clinical course [11,15,20,26,35,36]. A meta-analysis by Dadhania et al. [15] in a group of 937 UCa showed that muscle-invasive basal bladder cancers had shorter survival compared to luminal cancers.

In our study, in a multivariate analysis of overall survival, a higher probability of CK5/6(+) recurrence was a significant independent factor associated with shorter OS. We demonstrated that patients with at least one CK5/6(−) recurrence had significantly longer OS compared to patients with only CK5/6(+) UCa recurrences. The estimated risk of death was 2.859× higher in patients with CK5/6(+)-only recurrences. The presence of CK5/6(+) expression in UCa recurrences is a marker of poor prognosis.

In contrast, a higher probability of recurrence with GATA3(+) expression was a significant independent factor associated with longer OS and an estimated 13-fold reduction in the risk of death. Similarly, in univariate analysis, the probability of UCa recurrence with CK5/6(+) and GATA3(+) expression had a significant impact on OS. A higher probability of recurrence with CK5/6(+) expression was associated with significantly shorter OS, while a higher probability of recurrence with GATA3(+) expression was associated with significantly longer OS. The presence of GATA3(+) expression in UCa recurrences was associated with better prognosis. According to the literature, the presence of GATA3 expression is a marker of proper cell differentiation [31,34,37,38]. Wang et al. demonstrated that among 78 patients who had not previously undergone chemotherapy, low GATA3 staining was associated with worse recurrence-free survival in both univariate and multivariate analyses [38].

We did not demonstrate an effect of CK5/6 and GATA3 expression in the primary tumor on OS. This is consistent with some previous reports [17,24,25,26,29].

Bejrananda et al. [23] in a study of 132 UCa cases after radical cystectomy showed the longest OS associated with the mixed subtype [23] and the best survival times in patients with GATA3 and CK5/6 expression [23]. Patients with double-negative tumors had the worst OS [23].

A study by Kollberg et al. [25] in a group of 397 patients undergoing cystectomy showed no association of UCa subtypes with survival. Similarly, Weyerer et al. showed no association of disease-specific survival and recurrence-free survival in patients with MIBC with molecular subtypes [39].

In previous studies, the prognostic significance of GATA3 and CK5/6 was controversial [25,40,41]. Some publications indicate that loss of GATA3 expression leads to UCa progression [37,40]. Miyamoto et al. reported that loss of GATA3 expression predicted poor prognosis for patients with MIBC [40]. Loss of GATA binding protein 3 was associated with high-grade and/or muscle-invasive tumors, whereas strong expression was an independent predictor of poor prognosis [40]. Other studies showed that GATA3 expression had no significant influence on either cancer-specific survival or progression-free survival (PFS) [25,41]. Decreased GATA3 expression is associated with the promotion of VEGF (vascular endothelial growth factor) and MMP-2 and MMP-9 (matrix metalloproteinase 2 and 9), which initiate tumor progression, enhance angiogenesis, and promote the development of cancer metastasis [40,42].

A prospective study conducted by Rashad et al. [43] in a group of 50 patients with MIBC showed that GATA3-positive, luminal, and double-positive subtypes tend to have longer OS and PFS. A positive CK5/6 result had no impact on treatment outcome [43].

Calvet et al. [44] described the association of the expression of Cancer-Associated Fibroblasts (FAPs) with a more advanced stage of UCa and loss of expression of luminal markers, including GATA3. Expression of FAP and basal markers CK5/6 and CD44 was associated with significantly shorter disease-specific survival [44].

In our study, we found no correlation with sex for either CK5/6 or GATA3 expression. In contrast, Sangendoulce et al. demonstrated that significantly higher expression of CK5/6(+) and GATA3(+) occurred in men [29]. The authors also showed that higher CK5/6 expression together with loss of GATA3 expression were associated with infiltration of the muscularis propria [29].

Our results may have been influenced by molecular changes in UCa recurrences associated with intra-tumor heterogeneity of the primary UCa, as well as changes related to interactions with the microenvironment in two anatomical sites and the chemotherapy and radiotherapy used [19,20]. It is also important to note that our study population included both NMIBC and MIBC. Available publications on urothelial carcinoma often include patients at various stages of tumor. The histopathological material analyzed included surgical samples, as well as small sections taken during cystoscopy, which can yield false-negative results [23]. Large studies and meta-analyses related to the new UCa classifications have mostly been conducted in MIBC ≥T2 [17,24,29,40,45]. However, some studies included patients with NMIBC tumors in their subgroups [15,37,46]. NMIBCs are not a homogeneous group and include NMIBC low-grade papillary carcinomas and NMIBC high-grade (HG) bladder cancers containing flat urothelial CIS and pTaHG lesions. It is emphasized that a luminal-like phenotype has been correlated with unfavorable patient outcomes in NMIBC [47,48,49] and non-infiltrating urothelial papillary cancer of the upper tract [50,51], in contrast to observations made in MIBC [34].

In the present study, no significant association of tumor stage with overall survival was found. This may be related to the small group of patients, as well as the presence of samples taken via cystoscopy with tumor size designation of Tx. Available reports emphasize that approximately 50% of patients with locally advanced disease are underestimated [2,3,4].

In univariate analysis, surgical treatment was associated with significantly shorter DFS-1. This may be related to patients with a diagnosis of at least pT1 G3 cancer being qualified for surgery; hence, the prognosis for recurrence and survival was worse than for less advanced (NMIBC) tumors at the moment of diagnosis. The use of neoadjuvant chemotherapy was associated with a significant prolongation of DFS. This is consistent with reports in the literature [3,7].

In our study, there were 3.5 times more diagnoses of urothelial carcinoma in men than in women. This is consistent with data from the literature [4,5,15,17,20,24,29]. The mean age of diagnosis was 68 years (min. 49–max. 86 years). This is also consistent with literature data on Caucasians [4,44,52]. A relatively younger age of diagnosis is observed in countries with a higher incidence of squamous cell carcinoma [23,37].

In the present study, the most common UCa subtype in the primary tumors (64.9%) and in recurrences was the luminal subtype, followed by the mixed subtype (30% of primary tumors) and basal carcinomas (2.5% of primary tumors). Similar results were also obtained by other researchers [31,37,44,45]. In contrast, a mixed subtype predominated in the studies of Rozzaghdoust and Wang (43–48% and 48.4%, respectively) [24,38]. These discrepancies may be due to ethnic differences, differences in immunohistochemical assay methodology, antibodies used, or cutoff values for positive reactions.

The conducted study indicates the significance of GATA3 and CK5/6 evaluation both in the primary UCa and in recurrences. CK5/6 and GATA3 in urothelial carcinoma recurrences may differ from CK5/6 and GATA3 expression in primary UCa tumor. In everyday clinical practice, it is extremely important to identify patients with poor prognosis among a large group of patients with recurrent UCa. A simple, easily accessible screening test such as immunohistochemical staining could be helpful in conducting oncological surveillance in a group of patients with recurrent UC. This will allow for better clinical control and early, adequate oncological treatment. In patients with recurrent UCa CK5/6(+), close oncological surveillance is necessary.

The study presented above was retrospective in nature and involved a relatively small group of patients. We treat it as a pilot study. The results obtained require confirmation in a prospective study that also includes molecular testing and confirms the usefulness of such screening.

## 5. Conclusions

1. It is advisable to routinely determine CK5/6 and GATA3 in primary tumors and in UCa recurrences due to its prognostic significance. CK5/6 and GATA3 expression in urothelial carcinoma recurrence may differ from CK5/6 and GATA3 expression in primary UCa tumor.
2. Intensified oncological surveillance is suggested for patients with recurrent CK5/6(+) UCa. Patients with at least one UCa CK5/6(−) recurrence have better prognosis compared to patients with only CK5/6(+) recurrences.

## Figures and Tables

**Table 1:** Characteristics of the patients.

Parameter	*n* (%)
Number of patients *n* (%)	77 (100%)
Age (years)	
median = 68	
≤68	39 (50.7%)
>68	38 (49.3%)
Sex	
Female *n* (%)	17 (22.1%)
Male *n* (%)	60 (77.9%)
Tumor stage T *	
Ta	17 (22.1%)
T1	17 (22.1%)
T2	16 (20.8%)
T3	5 (6.5%)
Tx **	22 (28.5%)
Grade ***	
G1	7 (9.3%)
G2	23 (30.7%)
G3	45 (60.0%)
Subtype of UCa in the primary tumor	
Subtype 1: GATA 3+,CK 5/6-, luminal subtype	50 (64.9%)
Subtype 2: GATA-3−, CK 5/6+, basal subtype	2 (2.5%)
Subtype 3: GATA3+, CK 5/6+, mixed subtype	23 (30.0%)
Subtype 4: GATA3−, CK5/6-, double-negative subtype	2 (2.5%)
Surgery	
YES	35 (45.5%)
NO	42 (54.5%)
Chemotherapy	
YES	46 (59.7%)
NO	31 (40.3%)
Radiotherapy ****	
YES	18 (23.4%)
NO	57 (74.0%)

* T—tumor stage. ** In 22 cases, T was not assessed in the biopsy taken during cystoscopy (primary tumor). *** Grade—histological maturity. In two cases, Grade was not assessed in the primary UCa due to technical reasons. **** No data for 2 patients on whether radiotherapy was conducted.

**Table 2:** Characteristics of UCa recurrences. Immunohistochemical staining.

Number of Patients with Subsequent Recurrences	Number of Positive and Negative Immunohistochemical Staining Reactions			
	GATA3(+)	GATA3(−)	CK5/6(+)	CK5/6(−)
first recurrence *n* = 57	54	3	18	39
second recurrence *n* = 40	38	2	9	31
third recurrence *n* = 22	20	2	6	16
fourth recurrence *n* = 15	14	1	5	10
fifth recurrence *n* = 9	9		3	6
sixth recurrence *n* = 2	2		1	1
seventh recurrence *n* = 2	2			2
*n* = 147 (100%) *	139 (94.6%)	8 (5.4%)	42 (28.6%)	105 (71.4%)

* A total of 147 recurrences (100%) were identified: GATA3(+), 139 (94.6%); GATA3(−), 8 (5.4%); CK5/6(+), 42 (28.6%); CK5/6(−), 105 (71.4%).

**Table 3:** Characteristics of UCa recurrences. Grade.

UCa Recurrences	Grade (G) *n* (%) *		
*n*	G1	G2	G3
first recurrence *n* = 56 (100%)	13 (23.2%)	12 (21.4%)	31 (55.4%)
second recurrence *n* = 31 (100%)	8 (25.8%)	9 (29.0%)	14 (45.2%)
third recurrence *n* = 14 (100%)	5 (35.7%)	6 (42.8%)	3 (21.4%)
fourth recurrence *n* = 11 (100%)	6 (54.5%)	1 (9.1%)	4 (36.4%)
fifth recurrence *n* = 6 (100%)	2 (33.3%)	1 (16.7%)	3 (50.0%)
sixth recurrence *n* = 2 (100%)	2 (100.0%)		

* *n* (%)—number of patients with grading in the histopathological samples (percentage of patients). ** in the case of two patients diagnosed with a seventh relapse, for technical reasons, the grade was not evaluated.

**Table 4:** Evaluation of the association of UCa CK5/6(−) and CK5/6(+) expression in the primary tumor with age at diagnosis and the probability of recurrence without CK5/6(−) expression and recurrence with CK5/6(+) expression. Mann–Whitney U test.

Variable	Primary Tumor CK5/6(−)	Primary Tumor CK5/6(+)	*p*
	Medium ± SD * (Min–Max)	Medium ± SD * (Min–Max)	
Age	70.15 ± 8.04 (50.00–86.00)	66.20 ± 8.25 (49.00–86.00)	0.046
probability of recurrence CK5/6(−)	0.84 ± 0.26 (0.00–1.00)	0.27 ± 0.41 (0.00–1.00)	0.000005
probability of recurrence CK5/6(+)	0.15 ± 0.26 (0.00–1.00)	0.73 ± 0.41 (0.00–1.00)	0.000005

*p* < 0.05 was considered significant. * SD—standard deviation.

**Table 5:** Disease-free survival **(DFS-1)** and relative hazard of recurrence. Univariate analysis.

		DFS-1 *	
Independent Variable	*n*	Hazard Ratio (95%CI)	*p*
Grade ** (/1 grade)	75	1.335 (0.831–2.146)	0.232
T *** (/1 grade)	55	1.379 (0.985–1.931)	0.061
GATA3(+) expression primary tumor (yes vs. no)	77	0.327 (0.097–1.100)	0.071
CK5/6(+) expression primary tumor (yes vs. no)	77	1.396 (0.735–2.651)	0.307
Chemotherapy (yes vs. no)	77	0.741 (0.383–1.433)	0.373
Radiotherapy (yes vs. no)	77	0.671 (0.318–1.415)	0.294
Sex (M vs. F)	77	1.363 (0.570–3.256)	0.486
Age (/1 year of life)	77	1.004 (0.964–1.044)	0.855
Surgery (yes vs. no)	77	2.483 (1.309–4.711)	0.005
Probability of recurrence CK5/6(+)	58	1.383 (0.663–2.883)	0.386
Probability of recurrence GATA 3(+)	58	0.374 (0.131–1.073)	0.067
Neoadjuvant chemotherapy	71	0.498 (0.256–0.971)	0.04

Taken as significant, *p* < 0.05; *n* = number of patients. * DFS-1—time from first diagnosis to recurrence ≥T1. ** Grade—histological maturity of the tumor at the time of diagnosis. *** T—tumor size at the time of diagnosis.

**Table 6:** Overall survival (OS). Univariate analysis.

		OS *	
Independent Variable	*n*	Hazard Ratio (95%CI)	*p*
Grade ** (/1 grade)	75	1.607 (0.944–2.735)	0.080
T *** (/1 grade)	55	1.032 (0.720–1.490)	0.862
GATA3(+) expression in primary tumor (yes vs. no)	72	0.731 (0.0221–2.422)	0.609
CK5/6(+) expression in primary tumor (yes vs. no)	72	1.772 (0.913–3.440)	0.098
Chemotherapy (yes vs. no)	77	1.420 (0.667–3.023)	0.363
Radiotherapy (yes vs. no)	77	1.081 (0.554–2.109)	0.819
Sex (M vs. F)	77	1.258 (0.551–2.872)	0.585
Age (/1 year of life)	77	1.004 (0.964–1.046)	0.833
Surgery (yes vs. no)	77	1.470 (0.769–2.809)	0.243
Probability of recurrence CK5/6(+)	58	3.586 (1.499–8.571)	0.004
Probability of recurrence GATA3(+)	58	0.260 (0.079–0.853)	0.026
Neoadjuvant chemotherapy	71	0.982 (0.488–2.018)	0.982

*p* < 0.05 was considered significant; *n*—number of patients. * OS—time from first cancer diagnosis to death or last follow-up. ** Grade—histological maturity of primary UCa. *** T—tumor stage at the time of diagnosis.

**Table 7:** Overall survival (OS). Multivariate analysis (*n* = 43).

Independent Variable (*n* = 43)	OS *	
	Hazard Ratio (95%CI)	*p*
Age (/1 year of life)	0.960 (0.897–1.02)	0.174
Surgery (yes vs. no)	1.059 (0.379–2.965)	0.911
Grade ** (/1 grade)	2.279 (0.919–5.659)	0.075
T *** (/1 grade)	0.765 (0.421–1.391)	0.380
Probability of recurrence GATA3(+)	0.0784 (0.010–0.609)	0.015
Probability of recurrence CK5/6(+)	2.859 (1.028–7.949)	0.044

*p* < 0.05 was considered significant. * OS—time from first cancer diagnosis to death or last follow-up. ** Grade—histological maturity of the primary UCa. *** T—tumor stage of the primary UCa.

## Data Availability

The material will be available to interested researchers upon request.
